# Human tendon-on-a-chip for modeling vascular inflammatory fibrosis

**DOI:** 10.21203/rs.3.rs-3722255/v1

**Published:** 2023-12-13

**Authors:** Hani Awad, Raquel Ajalik, Rahul Alenchery, Isabelle Linares, Terry Wright, Benjamin Miller, James McGrath

**Affiliations:** University of Rochester; University of Rochester; University of Rochester; University of Rochester; University of Rochester; University of Rochester; University of Rochester

**Keywords:** Vascular inflammation, myofibroblasts, fibrosis, Tendon-on-a-Chip, adhesions, peritendinous injury, fibrotic conditions, human microphysiological model

## Abstract

Understanding vascular inflammation and myofibroblast crosstalk is critical to developing therapies for fibrotic diseases. Here we report the development of a novel human Tendon-on-a-Chip (hToC) to model this crosstalk in peritendinous adhesions, a debilitating fibrotic condition affecting flexor tendon, which currently lacks biological therapies. The hToC enables cellular and paracrine interactions between a vascular compartment harboring endothelial cells and monocytes with a tissue hydrogel compartment containing tendon fibroblasts and macrophages. We find that the hToC replicates in vivo inflammatory and fibrotic phenotypes in preclinical and clinical samples, including myofibroblast differentiation and tissue contraction, excessive ECM deposition, and inflammatory cytokines secretion. We further show that the fibrotic phenotypes are driven by the transmigration of monocytes from the vascular to the tissue compartments of the chip. We demonstrate significant overlap in fibrotic transcriptional signatures in the hToC with human tenolysis samples, including mTOR signaling, a regulatory nexus of fibrosis across various organs. Treatment with rapamycin suppressed the fibrotic phenotype on the hToC, which validates the hToC as a preclinical alternative for investigating fibrosis and testing therapeutics.

Fibrosis affects various tissues and organs of the body as an outcome of chronic inflammation. Chronic liver disease^1^, kidney^2^, lung^3^, and cardiac^4^ fibrosis, and acute or chronic injury^5^ are examples of fibrotic disorders, where inflammation leads to excess extracellular matrix (ECM) accumulation of a scar that replaces functional tissue. Peritendinous adhesions can develop in tendons due to chronic conditions like tendinopathy or acute injuries. Since tendons transmit muscle force and facilitate joint movements, fibrotic changes can make them prone to repeated injury and re-ruptures ^6^. The activation of myofibroblasts is a robust marker of peritendinous adhesions and other fibrosis pathologies. Myofibroblasts are activated by TGF-β1, a pleiotropic growth factor released during healing by numerous cells, including leukocytes, fibroblasts, and endothelial cells^7^. These cells proliferate excessively, produce scar tissue ECM, and contribute to the pathological feedback loop that amplifies TGF-β1 pro-fibrotic effects. Despite its pervasive role in chronic diseases, therapies to inhibit, attenuate, or reverse myofibroblast activation remain an unmet clinical need.

Until recently, the role of neovascularization in peritendinous adhesions was not fully appreciated or studied. Our own research and others have shown that increased vascularization is associated with tendinopathy^7–9^. Endothelial cells, which line the luminal surfaces of blood vessels, play several roles in fibrosis, both directly and indirectly, including the production of inflammatory and pro-fibrotic mediators that stimulate myofibroblast activation and leukocyte migration into damaged tissues^10^.

Progress in understanding the pathogenesis of fibrosis and in developing therapies has relied on mechanistic insights from animal models that do not always reliably translate to human disease^11^. In vitro disease models using human cells are essential complements to animal models and enable reductionist studies to reveal the molecular and cellular interactions between different cells that drive the fibrotic microenvironment^12,13^. Recent advances in liver, gut, and kidney microphysiological systems (MPS), to name a few examples, highlight the modernization of in vitro models for drug testing of complex diseases ^14^. Of particular value are vitro models of blood-tissue interfaces that capture the dynamic vascular and immune responses driving inflammatory diseases such as fibrosis ^15^. In this context, we seek a model of the injured human tendon, including its interaction with the vasculature and immune system, as a platform for discovering therapies to treat tendinopathies.

In this study, we develop and evaluate a human Tendon-on-a-Chip (hToC) model of inflammatory vascular fibrosis. The hToC platform is a modular device featuring a ‘vascular compartment’ with endothelial cells and monocytes and a ‘tissue compartment’ featuring a collagen-based ECM with tenocytes and resident macrophages. The hToC modularity enables the compartments to be prepared separately and then brought together to study the crosstalk between vascular endothelial cells, leukocytes, and tissue fibroblasts in response to pro-inflammatory and reparative factors such as TGF-β1.

Our results demonstrate that the fibrotic and inflammatory phenotypes observed in vivo also develop in the hToC, similar to tendon injury responses observed in mouse and human tissues. Further, we validate the transcriptional profile in the hToC against data derived from human tenolysis tissues ^16^. Notably, gene expression analysis reveals the activation of mTOR signaling as a consensus transcriptional signature of fibrosis in the hToC and the human tenolysis tissues. Given that mTOR signaling has recently emerged as a druggable target in the treatment of major organ fibrosis, which was also observed in the human tenolysis tissues and the hToC, we tested the therapeutic efficacy of mTOR inhibition in mitigating the fibrotic traits in the hToC. We found that a single dose of rapamycin suppressed myofibroblast activation and attenuated the mTOR activation in the vascular compartment, although it did not fully resolve the inflammatory secretome. These findings underscore the potential of the hToC as a model of the tendon injury microenvironment and, more broadly, as a platform for investigating the vascular-inflammatory crosstalk in fibrosis.

## Results

### A Modular Tendon-Vascular Interface Model

**Figure 1:**
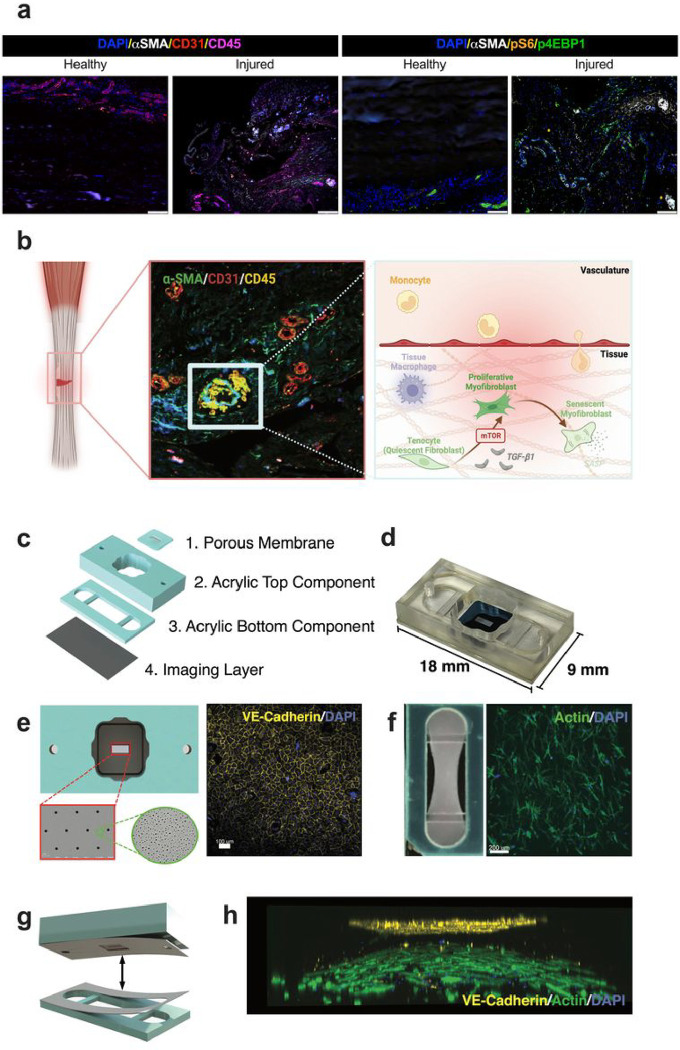
Design of the human Tendon-on-a-Chip (hToC). **a,** Micrographs of the injured human tendon with immunohistochemistry of fibrovascular scar depict the microenvironment modeled in the hToC through cellular and molecular interactions between the vascular and tendon tissue compartments shown in panel **b**. **c,** Exploded view of the fully assembled hToC device shown in panel **d**. **e,** The chip embedded in the top compartment features a Dual-Scale (DS) porous membrane with 5 μm pores (red outline) dispersed over a nanoporous (≤100nm pores) background (green outline), onto which ECs are cultured to form a cohesive vascular barrier with developed junction proteins (VE-Cadherin (yellow)). **f,** A tenocyte-embedded collagen construct is placed in the bottom compartment where bulk contraction of the hydrogel is induced by anchoring through two horizontal bars (left). F-actin staining shows fibroblastic cell morphology of tenocytes embedded in the collagen hydrogel. **g,** Modular design of the hToC allows for reversible binding of top and bottom compartments, creating a model of a vascular barrier in proximity to a tendon construct in the bottom channel as imaged in **h**.

While a healthy tendon is hypovascular, neovascularization is frequently observed in tendinopathy and scar tissue following acute injury and is considered a hallmark of chronic pathology. Traditionally, the role of vascularization in acute tendon injury and fibrotic peritendinous adhesions has been underemphasized, with the assumption that its primary purpose is to facilitate the arrival of immune cells and inflammatory factors to the injury site ^17,18^. In human tenolysis samples, we have observed notable spatial colocalization of neovascularization with circulating and tissue-infiltrating leukocytes in proximity to myofibroblast. Furthermore, this spatial localization was associated with increased activation of downstream mTOR effector proteins S6 and 4EBP1, which suggests a potentially aberrant cellular and molecular crosstalk involving mTOR signaling in the injury milieu (Fig. 1a). We postulated that these interactions at the tendon-vascular interface play a pivotal role in the pathogenesis of fibrosis and might offer novel therapeutic targets to attenuate fibrotic progression.

To model the tendon-vascular interface (Fig. 1b), we customized a two-compartment vascular barrier model^19^ to be integrated with a tendon tissue hydrogel (Fig. 1c,d).

Briefly, the hToC is composed of two custom-fabricated components: Component 1 is an acrylic block component featuring a 100 μL well with two full-thickness fluidic ports to access the bottom component and a bottom ledge lined with a pressure-sensitive adhesive (PSA) to seal to a membrane chip (Fig. 1e). The membrane chip contains an ultrathin (~ 100 nm thick) optically clear, porous silicon nitride membrane patterned in a 700 μm × 2 mm window^20,21^. The membrane features dual-scale porosity with 5 μm pores superimposed on a nanoporous silicon nitride (NPN) background (Fig. 1e). The 5 μm pores provide portals for cell transmigration from the vascular to the tendon hydrogel compartment, while the nanopores (~ 60 nm diameter; ~ 15% porosity) allow unhindered paracrine signaling by small molecule exchange throughout the membrane interface between compartments. Component 2 features a fluidic channel into which a fibroblast-laden collagen hydrogel (~ 65 μL) can be cast, with horizontal anchors suspended on either end of the channel (Fig. 1c,f) to constrain the fibroblast-mediated hydrogel axial contraction while permitting lateral contraction. A double-sided PSA lining on the top surface of Component 2 facilitates bonding to Component 1 (Fig. 1g) during the assembly of the full hToC (Fig. 1d).

Human umbilical vein endothelial cells (EC) were seeded onto the porous membrane to form the vascular interface monolayer (Fig. 1e). Human primary tendon fibroblasts (TC), isolated from tissues obtained from hand surgery were embedded in a TeleCol^®^-3 collagen hydrogel (500,000 cells/ml) and pipetted into Component 2 channel (Fig. 1f). The addition of tissue-resident macrophages (tMφ) into the tissue hydrogel and circulating monocytes (cMφ) into the vascular well simulates the leukocyte interactions in the vascularized injury milieu as observed in Fig. 1b.

**Figure 2:**
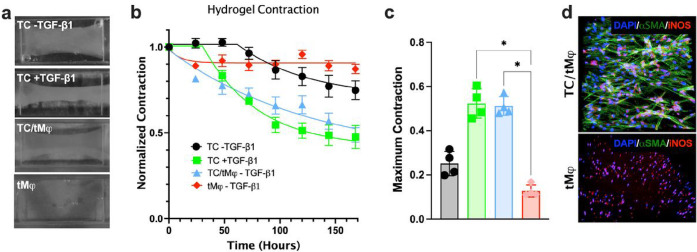
Tissue hydrogel contraction in the hToC. **a,** Hydrogel tissue contraction images illustrating distinct conditions: TC hydrogel monocultures without or with exogenous TGF-β1 (10 ng/ml), TC/tMj co-cultures without TGF-β1, and tMj hydrogel monocultures in the absence of TGF-β1. **b,** Hydrogel tissue contraction kinetics normalized to day 0. **c,** Maximum contraction of the hydrogel expressed as the ratio of the final to original hydrogel area. D, Immunofluorescence of myofibroblasts ( -SMA) and leukocytes (iNOS) in the representative hydrogels. Data is plotted as mean and standard deviation from 3 – 6 devices.

The modular design of the hToC enables the independent culture of the vascular and tissue compartments before their integration as a composite tissue (Fig. 2a). This feature is critical given the different culture conditions and times required for the EC monolayers to achieve confluent barrier function (24 hours) and for the differentiation and maturation of monocytes to tMφ in the hydrogel (6 days). By accommodating cell-specific timelines and requirements, the hToC can be used to study different cell combinations, including monocultures (TC only), cocultures (TC/EC or TC/tMφ), tricultures (TC/EC/CMφ or TC/tMφ/EC), or quad cultures (TC/tMφ/EC/cMφ) (Extended Fig. 1). Careful selection of device materials and layer thicknesses enables live microscopy and multiplex fluorescent imaging of cellular and molecular interactions in situ. The fluidic ports enable media sampling for cytokine analysis.

### Modeling Inflammatory-Fibrovascular Scar Tissue

Using a quad-culture protocol in the hToC, we modeled the injured tendon microenvironment involving endothelial cells, leukocytes, and tendon fibroblasts. Monocytes isolated from human peripheral blood donors were embedded and co-cultured with tenocytes (TC) in TeleCol^®^-3 hydrogel for a total cell concentration of 500 cells/μl at a fibroblast-to-monocyte ratio of 7:1^22^. We then differentiated the hydrogel-embedded monocytes into macrophages (tMφ) in Lonza’s XVIVO-10^™^ serum-free media supplemented with monocyte-colony stimulating factor (M-CSF) over six days. In the TC-laden hydrogel monoculture, treatment with TGF-β1 over seven days in culture increased hydrogel contraction to ~ 50% of its original area (Fig. 2a–c). Interestingly, hydrogel containing TC and tMφ also contracted to ~ 50% in the absence of TGF-β1, which was accompanied by the activation -SMA + myofibroblasts (Fig. 2d). The role of the tenocytes/myofibroblasts and leukocytes crosstalk in effecting the TC//tMφ hydrogel contraction was verified by the lack of contraction in the tMφ hydrogel monocultures. Together, this data suggests that even in the absence of TGF-β, tMφ secrete factors that lead to the activation of tenocytes into myofibroblasts, which subsequently contract the collagen hydrogel.

**Figure 3:**
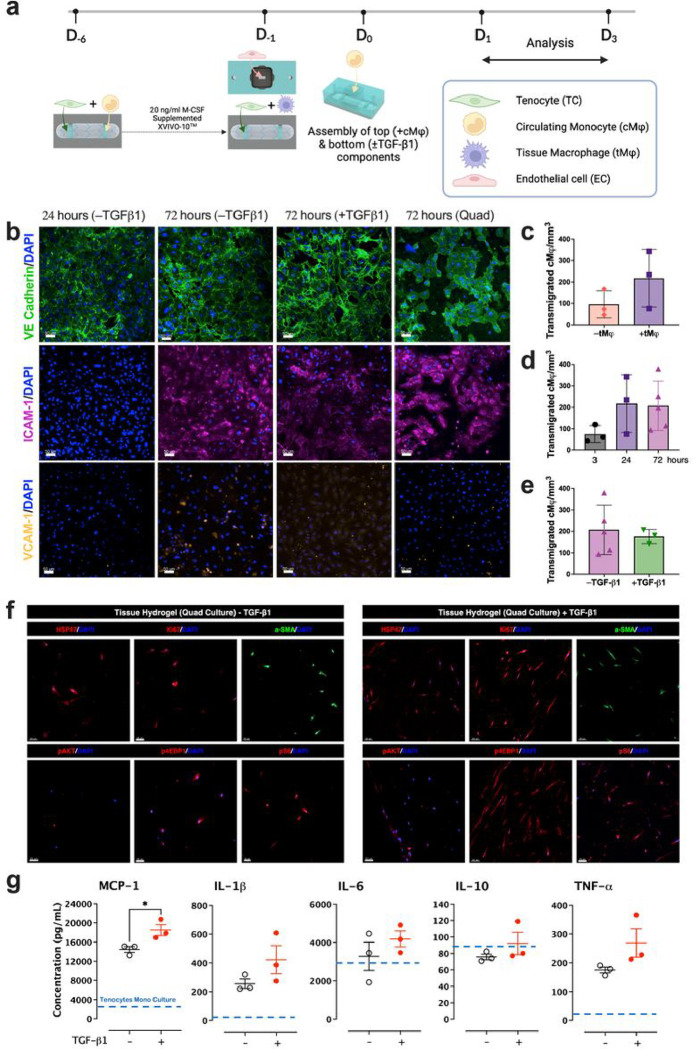
Modeling fibrosis and vascular inflammation in the hToC. **a**, A schematic of the experimental workflow for the hToC quad-culture assembly. **b,** The effect of the exogenous TGF-β1 treatment was examined on the EC mono culture stained for leukocyte adhesion and activation markers VE-cadherin **(green, row 1)**, ICAM-1**(purple, row 2)**, and VCAM-1**(yellow, row 3)** at 24- and 72-hours. Exogenous TGF-β1 was added directly to the luminal EC surface, incubated for 72-hours, and assessed with the same markers **(b, column 3)**. These results were compared to the EC monolayer removed from a 72-hour quad culture **(b, column 4)**. Scale bar = 50 mm. The transmigration of fluorescently labeled circulating monocytes (cM ) from the vascular compartment to the tissue hydrogel was quantified under various conditions, including **c,** tri cultures (without tM ) or quad cultures (with tM ), **d,** quad cultures without TGF-β1 over time, and **e**, quad cultures without or with exogenous TGF-β1 at 72 hours. **f,** Representative immunofluorescence sections of the tissue hydrogel in quad-cultures show fibroblastic morphology in tendon construct in the absence or presence of exogenous TGF-β1 treatment. Sections were stained for markers for collagen-specific molecular chaperone (HSP47), cellular proliferation (Ki67), myofibroblast activation (α-SMA), and mTOR pathway expression (pAKT, p4EBP1, pS6). **g,** The quad culture supernatants were tested from the combined vascular and tissue sides and sampled using a multiplex Luminex^®^ assay. The inflammatory cytokine secretory protein profile at 24 hours in the presence and absence of exogenous TGF-β1 (N=3–4) is displayed, where the dashed blue line represents secretion levels in monocultures (tenocytes-laden hydrogels) treated with TGF-β1. Scattered data presented with mean and standard deviation marks (n=3 devices). One-way ANOVA was used for comparison (*p < 0.05).

To create a vascular barrier, endothelial cells (EC) were plated on the membrane chip in the vascular compartment at 40,000 cells/mm^2^ and allowed to adhere to the membrane and reach confluence over 24 hours. To mimic circulating monocytes (cMφ) infiltration to the injury site through the vasculature, freshly isolated monocytes were introduced into the vascular compartment at 10,000 cells per 100 μL of XVIVO-10^™^ serum-free media (day 0). The two compartments were then assembled to create a quad culture system (TC/tMφ/EC/cMφ), which was treated with TGF-β1 (10 ng/ml) or vehicle, directly administered to the tendon hydrogel compartment for 24–72 hours (Fig. 3a).

When cultured separately, the vascular compartment exhibited robust activation of inflamed endothelium at 72 hours independent of the luminal exposure to TGF- β1 (Fig. 3b). This activation was manifested by the positive staining of intercellular adhesion molecule-1 (ICAM-1) and vascular cell adhesion molecule-1 (VCAM-1) and noticeable gaps resulting in a leaky vascular monolayer. Introducing TGF-β1 from the EC abluminal side in quad culture conditions resulted in comparable, robust vascular activation. Furthermore, quantification of the transmigration of fluorescently labeled cMφ through the vascular endothelium into the tissue hydrogel demonstrated the critical role of tMφ in stimulating the transmigration behavior, even in the absence of TGF-β1 treatment (Fig. 3c). Interestingly, there were no significant increases in transmigrated cMφ after 24 hours in quad cultures without TGF-β1 treatment (Fig. 3d). Finally, treatment with TGF-β1 in the quad cultures did not significantly increase the number of transmigrated cMφ after 24 hours (Fig. 3e).

Under the quad culture conditions, treatment with TGF-β1 led to an increased abundance of markers of fibrosis, including α-smooth muscle actin (α-SMA^+^) myofibroblasts, with characteristic contractile morphology, HSP47, a collagen-binding glycoprotein chaperone in the endoplasmic reticulum (ER), and Ki67, a marker of proliferation (Fig. 3f). Furthermore, treatment with TGF-β1 increased activation of the mammalian target of rapamycin (mTOR) signaling, evidenced by increased abundance of phosphorylated 4EBP1 and P70S6 (Fig. 3f), consistent with the observed upregulation of mTOR effector proteins p4EBP1 and pS6 in the vicinity of myofibroblasts in tenolysis tissue (Fig. 1a).

Previous experiments in mice have shown that several cytokines are systemically upregulated early and sustained over time following acute tendon injury^7^. We probed for eight of these secreted cytokines in the hToC culture supernatant using a Luminex detection assay at 24 and 72 hrs (Extended Fig. 2). MCP-1, IL-1β, IL-6, IL-10, and TNF- were significantly increased in quad-cultures at 24 and 72 hours compared to the monoculture of tenocyte-laden hydrogel (Fig. 3g). However, TGF-β1 treatment did not significantly affect their levels in the quad culture hToC. These results underscore the significance of cellular crosstalk of TC, tMφ, cMφ, and EC in driving the inflammation and TGF-β1 induced myofibroblast activation and mTOR signaling within the quad culture model, which could serve as markers of tendon fibrosis in the hToC.

### Validating the hToC as a Peritendinous Fibrosis Model

**Figure 4:**
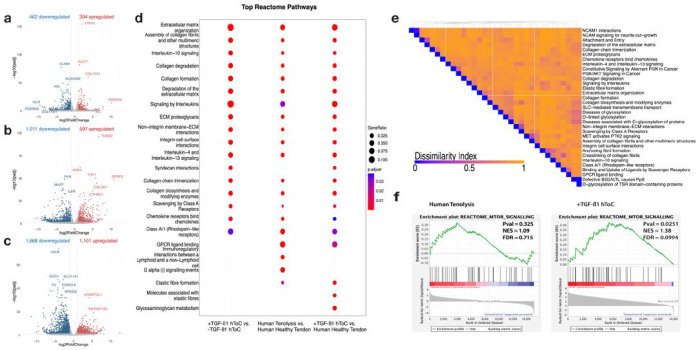
Validating the hToC as a Peritendinous Fibrosis Model. Volcano plots summarizing differential expression comparisons using DESeq2 of **a,** TGF-β1 treated versus untreated hToC, **b,** TGF-β1 treated hToC versus healthy human flexor tendon, and **c,** human tenolysis tissue versus healthy human flexor tendon. **d,** Dotplot graph identifies overlapping Reactome pathways, which are significantly enriched in the three comparisons, **e,** Dissimilarity matrix of Reactome pathways enriched in TGF-β1 treated hToC and Human tenolysis tissue relative to healthy human tendon. **f,** GSEA enrichment plots for Reactome MTOR Signaling Pathway in tenolysis tissue and TGF-b1 treated hToC. All DEG were based on P_adj_ < 0.05 and abs(Log_2_(FC)) > 1. N = 3 hToC devices per condition representing three biological replicates.

To validate the hToC quad culture as an in vitro model of vascular-inflammatory fibrosis, we performed bulk RNA-sequencing and benchmarked the results against published gene expression data from human tenolysis tissue obtained 2–3 weeks after tendon injury and healthy flexor tendon tissue (GSE108933)^16^. We first assessed the effect of TGF-β1 by analyzing the differential gene expression in TGF-β1 treated (+ TGF-β1) hToC compared to control, untreated hToC (-TGF-β1). Based on a twofold change (± 2FC) and adjusted p-value < 0.05 differential expression criteria, TGF-β1 treatment resulted in 462 downregulated genes and 304 upregulated genes (Fig. 4a). Notably, the vascular component exhibited fewer than 70 differentially expressed genes (DEGs), indicating a more significant effect of TGF-β1 on the tendon hydrogel. Subsequently, we compared the gene expression profile of the in vitro disease model (TGF-β1^+^ hToC) to healthy human tendon, which identified 1,011 downregulated genes and 597 upregulated genes (Fig. 4b). In contrast, a total of 1,668 genes were downregulated and 1,101 were upregulated in human tenolysis tissue relative to healthy tendon tissue (Fig. 4c).

We performed pathway enrichment analyses based on the DEGs in these comparisons. To evaluate the fidelity of the hToC disease model in simulating vascular-inflammatory fibrosis, we identified the top significantly enriched Reactome pathways in the hToC and demonstrated significant overlap with the pathways enriched in the human tenolysis tissue, which encompassed biological processes related to ECM deposition and remodeling and inflammatory signaling (Fig. 4d). To further assess the congruence of gene expression in the overlapping Reactome pathways, a Jaccard dissimilarity heatmap was plotted based on Euclidian distance of the gene sets between the hToC and tenolysis tissue (Fig. 4e). Despite observing high dissimilarity indices over many of the shared pathways, we observed significant congruence in key ECM, inflammation, interleukin signaling, including the PI3K/AKT (mTOR) signaling pathways. Additionally, gene set enrichment analysis (GSEA) revealed that the mTOR signaling pathway was significantly enriched in the hToC upon treatment with TGF-β1 (Fig. 4f).

### Rapamycin Effects on mTOR Signaling and Fibrotic Responses in hToC

**Figure 5:**
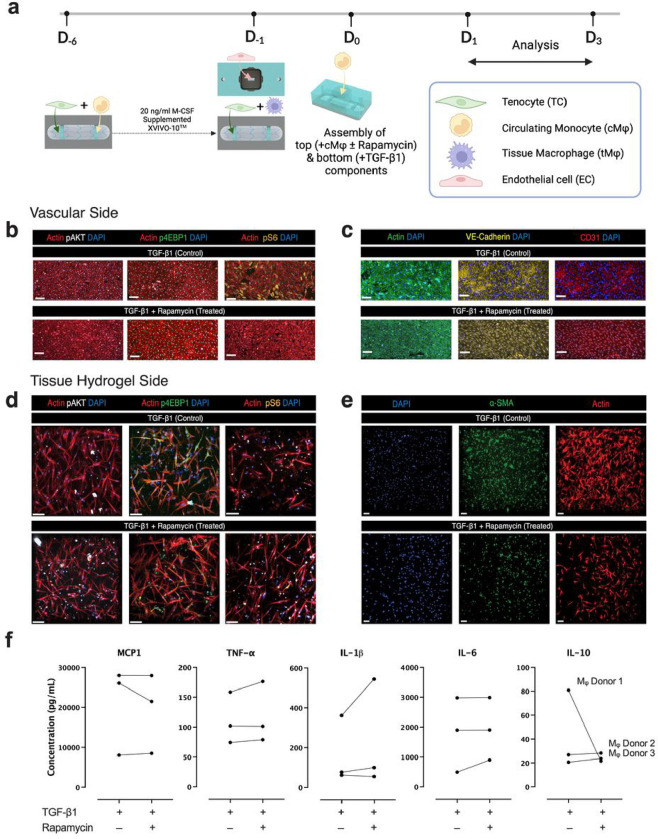
Rapamycin Treatment in the hToC. **a,** Schematic detailing quad-culture time course with 10 ng/ml TGF-β1 insult in the hydrogel compartment and 10 ng/ml rapamycin treatment into the vascular compartment on D_0_, followed by a 24-hour culture. Immunofluorescence staining of **b,** vascular monolayer and **d,** tissue construct for phosphorylated mTOR proteins pAKT (white), p4EBP1 (green), pS6 (orange) with F-actin counterstain (red). **c,** Immunofluorescence staining of the vascular side for endothelial junction protein VE-Cadherin (yellow), EC marker CD31 (red), and F-actin counterstain (green). **e,** Immunofluorescence staining of the tissue hydrogel for myofibroblast marker a-SMA and F-actin counterstain (red). **f,** Cytokine secretion analysis in the hToC top well and bottom compartment supernatant at 24 hours post-treatment was quantified using a LuminexÒ assay for pro-inflammatory cytokines. Each dot represents the mean of 3 technical replicates for three distinct cMj donors.

Given the observed involvement of mTOR in tendon adhesions in vivo, which is also recapitulated in the hToC, we then investigated the effects of rapamycin, an mTOR kinase inhibitor and FDA-approved drug^23^, as a treatment to mitigate the fibrosis phenotype in the hToC. In these experiments, we added 10 ng/ml of rapamycin to the culture media at the time of TGF-β1 and cMφ introduction (D_0_) to the quad culture hToC (Fig. 5a). The dose was selected based on measured plasma drug concentration in clinical studies^23^. We evaluated rapamycin’s effects on mTOR signaling, vascular inflammation, and the activation of myofibroblasts in the hToC. While treatment with rapamycin exerted negligible effects on pAKT and p4EBP1 activation in the endothelial cells, it significantly inhibited pS6 activation in the vascular endothelium (Fig. 5b). Rapamycin induced a uniform and more evenly distributed expression of junctional VE-cadherin and mitigated the noticeable gaps in the leaky vascular monolayer that are prominent in the TGF-β1 control (Fig. 5c).

In the tissue compartment of the TGF-β1 control, p4EBP1 expression was not limited to fibroblastic cells but was also observed in leukocytes (tMφ and infiltrating cMφ) (Fig. 5d). Upon treatment with rapamycin, the expression of p4EBP1 decreased in tendon fibroblasts but not in leukocytes. Treatment with rapamycin showed no discernable effects on pS6 in the tendon fibroblasts (Fig. 4d). However, the expression of α-SMA in the tendon fibroblasts significantly decreased and was accompanied by remarkable morphological changes such that the number of enlarged and irregularly shaped myofibroblasts with prominent stress fibers was reduced (Fig. 5e). These results collectively demonstrate that rapamycin treatment in the hToC model had significant effects in both the vascular and tissue compartments, attenuating the effects of TGF-β1 on the vascular endothelium and the myofibroblast phenotype.

Three monocyte donors were used to provide tMφ and cMφ to account for patient variability in the experimental design. To monitor changes in the inflammatory and fibrotic response to rapamycin, secreted inflammatory cytokines were evaluated after 24 hours of treatment with a Luminex^®^ assay for each distinct donor. While no significant rapamycin-induced changes were observed from controls on average, the secreted inflammatory cytokine levels varied significantly between donors (Fig. 5f). This finding underscores the importance of using multiple donors in the assessment of drug safety and efficacy in pre-clinical studies with MPS platforms and demonstrates the hToC’s sensitivity to capture the expected patient variability.

**Figure 6:**
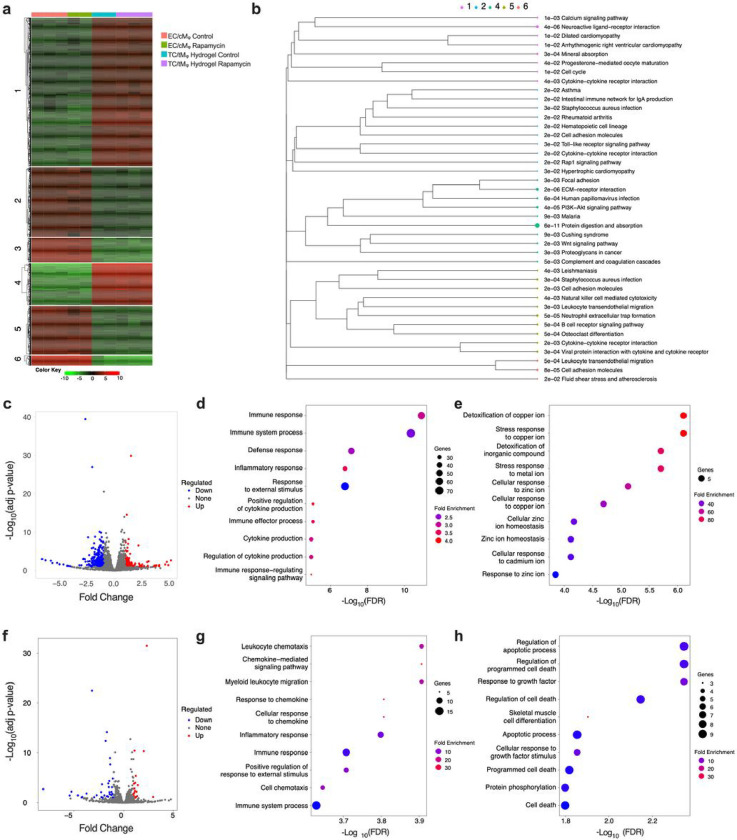
Transcriptomic Analysis of hToC Under Rapamycin Treatment. **a,** Representative heatmap of the top 2000 genes in the hToC, revealing distinct transcriptional patterns between the vascular and tissue compartments. **b,** Enrichment of Gene Ontology (GO) Biological Process (BP) terms represented in the hToC. **c,** Volcano plot illustrating gene expression changes in the vascular (EC/cMφ) compartment due to rapamycin treatment. **d,** GO BP terms related to downregulated genes in the vascular compartment. **e,** GO BP terms associated with upregulated genes in the vascular compartment. **f,** Volcano plot showing gene expression changes in the tissue hydrogel (TC/tMφ) compartment post-rapamycin treatment. **g,** GO BP terms linked to downregulated genes in the tissue hydrogel compartment. **h,** GO BP terms associated with upregulated genes in the tissue hydrogel compartment.

To gain further insights into the effects of rapamycin on the regulation of the inflammatory and fibrotic response, bulk RNA-seq analysis of the hToC was carried out for each of the three different M donors (rapamycin compared to control) using the integrated Differential Expression & Pathway (iDEP1.12) web application^24^. A representative heatmap of the top 2000 differentially expressed genes in the hToC (Fig. 6a) shows distinct transcriptional clustering and the associated enrichment of Gene Ontology (GO) Biological Processes (BP) (Fig. 6b) terms. In general, GO terms related to inflammation, ECM remodeling, cell cycle, cell adhesion and transmigration, and various signaling pathways, including the PI3K-Akt pathway, were enriched for all donors. Differential gene expression analysis revealed that depending on the donor, rapamycin treatment resulted in 238–452 downregulated genes and 86–248 upregulated genes in the vascular (EC/cMφ) compartment (Fig. 6c). In contrast, 15–35 genes were downregulated and 11–35 genes were upregulated in the tissue hydrogel (TC/tMφ) compartment (Fig. 6f). This underscores a predominant influence of rapamycin on the inflamed vascular endothelium, likely associated with the enhanced vascular barrier integrity observed post-rapamycin treatment (Fig. 5c), which is consistent with published reports of rapamycin’s cytoprotective effects against endothelial cell damage^25^. The GO BP terms enriched by DEGs in the vascular and tissue hydrogel compartments were then examined. In the vascular compartment, downregulated genes in rapamycin-treated devices enriched immune and defense responses pathways, including the regulation of cytokines and responses to external stimuli (Fig. 6d), and the upregulated genes enriched pathways regulating metal ion levels, detoxification, and stress responses (Fig. 6e). In the tissue hydrogel compartment, the downregulated genes in rapamycin-treated devices enriched recruitment of immune cells, chemokine signaling, and various immune responses (Fig. 6g), and upregulated genes enriched tissue damage and repair, including cell death and survival processes and responses to growth factors (Fig. 6h).

## Discussion

In this study, we engineered and characterized a novel human Tendon-on-a-Chip (hToC) platform to study inflammatory fibrosis. The hToC model recapitulates inflammatory and fibrotic characteristics observed in preclinical and clinical specimens. These characteristics include key markers of inflammatory fibrosis, such as myofibroblast differentiation, tissue contraction, excessive extracellular matrix (ECM) deposition, and the secretion of inflammatory cytokines.

Our data suggest that interactions between leukocytes and endothelial cells in the quad cultures are critical for promoting elevated inflammatory secretome and activating adhesion molecules that mediate leukocyte transendothelial migration, regardless of exogenous TGF-β1 treatment, which is consistent with established paradigms ^18,26^. Tendon fibroblasts, in turn, are affected by the secreted cytokines, which activate their differentiation into myofibroblasts, characterized by elevated collagen production and a proliferative and contractile state. The activation of myofibroblasts is induced by TGF-β1, a major inflammatory growth factor released during healing by neutrophils, macrophages, and T cells^27^. Macrophages are innate immune cells that accumulate at inflammatory fibrosis sites. The inflammatory signals from macrophages amplify fibrosis through various mechanisms, such as attracting more immune cells, inducing myofibroblast differentiation and cellular senescence, and impairing tissue remodeling^28^ through secreting a multitude of growth factors and cytokines, including TGF-β1^29^, leading to the persistence of an inflammatory response, ultimately culminating in chronic scar tissue. However, the role of angiogenesis in the development of tendon adhesions has been less appreciated until recently. Our research and others have shown increased angiogenesis in the formation of fibrotic adhesions in tendons^7,9,30^. Yet, the endothelial vasculature’s roles in fibrosis remain to be fully elucidated.

The hToC provides the first in vitro model of the myofibroblast microenvironment (MME), which we define to include the myofibroblast and the surrounding blood vessels, immune cells, tenocytes, signaling molecules, and the extracellular matrix (ECM). This is analogous to the tumor microenvironment (TME), defined as the cellular and molecular environment in which cancer cells reside and interact with surrounding non-cancerous cells, extracellular matrix, and signaling molecules ^31^. The invasiveness of cancer cells, including their propensity to metastasize and their resistance to cytotoxic drugs, is regulated by the TME. By analogy, we postulate that disrupting the MME therapeutically could resolve fibrotic pathologies. Therefore, the hToC will be a valuable new tool for delineating novel cellular interactions and signaling in the MME, which could lead to therapies to resolve peritendinous adhesions and, more broadly, chronic fibrosis.

We identified a substantial convergence in the transcriptional signatures associated with fibrosis between the hToC model and human tenolysis samples^16^. Specifically, we observed commonalities in the activation of the mTOR pathway. While a specific role for mTOR signaling in tendon injury and peritendinous adhesions has not been previously reported, it serves as a regulatory hub for fibrosis^32^. Tendon-specific Raptor/mTOR knockout mouse models have decreased tendon strength and elasticity, collagen fiber diameter, and paw grip strength, suggesting that mTORC1 signaling is a critical regulator of postnatal tendon development^33,34^. A recent genome-wide association study (GWAS) of susceptibility to idiopathic pulmonary fibrosis strongly implicated mTORC1 signaling as a causal driver of fibrosis pathobiology in humans^35^. Recent clinical trials demonstrated that short-term treatment with sirolimus, an mTOR inhibitor, led to a decrease in the levels of circulating fibrocytes in individuals with IPF, accompanied by an acceptable safety profile^36^.

Having identified mTOR activity as a driver of the fibrotic phenotype in the hToC, we then used the platform to explore the therapeutic potential of rapamycin. Rapamycin substantially impacted the vascular endothelium, as evidenced by the significant inhibition of pS6 activation and the promotion of a more uniform expression of junctional VE-cadherin. Moreover, it mitigated the distinctive gaps between endothelial cells in the vascular compartment following TGF-β1 treatment. The ability of rapamycin to improve the barrier function of the vasculature has been previously reported in neuroinflammatory diseases, in part by attenuating the inflammatory response^37,38^. In the tissue hydrogel, rapamycin reduced the expression of p4EBP1 in tendon fibroblasts and a notable decrease in α-SMA expression, indicating a modulation of the myofibroblast phenotype.

Additionally, bulk RNA-seq analysis provided insights into the transcriptional changes induced by rapamycin. Notably, rapamycin predominantly influenced the vascular endothelium, with many downregulated genes related to immune and defense responses and leukocyte transendothelial migration, corroborating in vivo observations^39^. Furthermore, rapamycin downregulated genes in the tissue hydrogel enriched immune cell recruitment and chemokine signaling while upregulated genes enriched tissue repair and cell survival mechanisms, consistent with previous findings^40^. In contrast, the single dose of rapamycin had a minimal impact on the inflammatory cytokine profile in the hToC, although the results depended on the donor used to populate resident and circulating monocytes in the model. Thus, while circulating and resident immune cells still produced an inflammatory secretome despite treatment with rapamycin, the endothelial cells, tenocytes, and myofibroblasts became more resistant to the inflammatory microenvironment.

This work is not without limitations. The interactions between monocytes and the vascular endothelium were evaluated under static (no flow) conditions. Introducing physiological fluid flow would allow for clearance of secreted cytokines and enable ECs to undergo shear priming and physiological conditioning^41^. Integrating fluid flow would also facilitate interactions between ECs and circulating leukocytes, which will be evaluated in future studies. Furthermore, while using primary cells is practical, it introduces donor variability and heterogeneity into the model, highlighted here by the variable cytokine secretion response with different monocyte donors. Such variable, difficult-to-control effects make primary cells undesirable for screening. To overcome this challenge, improved models of peritendinous injury could leverage induced pluripotent stem cells (iPSCs), which offer the advantage of enabling isogenic models and well-defined patient cohorts for simulated clinical trials.

In conclusion, this study introduces and characterizes a novel human Tendon-on-a-Chip (hToC), revealing its ability to recapitulate key features of vascular inflammatory fibrosis in injured tendons. Our results highlight previously underestimated contributions of vascular endothelial cells in the myofibroblast microenvironment and identify the mTOR pathway as a critical regulator of fibrosis in this context. Rapamycin shows promise in mitigating vascular endothelial dysfunction and myofibroblast activation in the hToC model, though its impact on inflammatory cytokines is modest and varies between donors. Thus, while our findings shed light on novel aspects of fibrovascular inflammatory myofibroblast microenvironment and demonstrate the potential of the hToC to evaluate potential therapeutic interventions, we acknowledge the need for future studies incorporating physiological fluid flow and the use of induced pluripotent stem cells to refine our understanding and personalize therapeutic screening.

## Methods

### Silicon Dual-Scale Porous Membranes

‘Dual-scale’ silicon nitride membranes were purchased from SiMPore Inc. (Rochester, NY). These are commercial versions of membranes first described by Salminen et al. (2019)^21^. The membrane is created by patterning a microporous array atop a nanoporous silicon nitride (NPN) background. The nanoporous background has an average nanopore size of 44.6 nm, a porosity of ~ 15%, and a membrane thickness of ~ 100 nm. We patterned micropores at 5 μm in diameter to enable monocyte transmigration from the vascular to tissue sides of the hToC. We limited the microporous density to produce a ~ 1% porosity (superimposed upon the nanoporous background to give a total of ~ 16% porosity). This density of micropores is well below the 10% value shown to give monolayer stability^30^ and does not hinder imaging due to light scattering by micropores.

### hToC Components

The top well component (top component) and bottom channel component (bottom component) of the hToC were manufactured at ALine Inc. (Signal Hill, CA) using laser cutting and lamination processes that are compatible with mid-volume production (hundreds to tens of thousands) of microfluidic components in a single production run. Parts were produced using a batch process and diced after final lamination for more reliable handling in the laboratory. The top well component contains fluidic access ports to the bottom channel that create sealed fits against P20/P200 pipette tips (VWR, 76323–390). Using pressure-sensitive adhesive (PSA), the top and bottom components were reversibly adhered to form the hToC device. Devices are free of any molded PDMS, and although they contain silicone as part of the adhesive layers, the material accounts for < 5% of the fluid-exposed surface. Thus, concerns about the loss of small molecules via absorptive loss^42^ are minimized in the hToC. The external surfaces of the shipped components include an additional protective layer (masking material) to maintain cleanliness and sterility during shipment and storage. The masking material is removed by the user before assembly of the components in a laminar floor hood as described in McCloskey et al. (2022)^19^.

### Tissue Formation and Maturation

#### Human Tenocyte Isolation & Culture

Tenocytes were isolated from tendon tissue fragments retrieved from hand surgery procedures at the University of Rochester Medical Center under an approved Institutional Review Board (IRB) protocol (STUDY00004840). The isolated tendon tissues were immersed in alpha minimum essential medium (αMEM) supplemented with 10% Pen-Strep (10,000 U/mL penicillin/10 mg/mL streptomycin) and cut into 1-mm^3^ pieces using ophthalmic scissors and a gentleMACS^™^ Dissociator (Miltenyl Biotec, 130–093-235). After mechanical digestion, the tissue was transferred to an enzyme solution consisting of 2.5mg/ml of Collagenase D (Millipore Sigma, 11088858001), 3mg/ml of Dispase II (Millipore Sigma, D4693), and 1mg/ml of DNase (New England Biolabs, M0303) dissolved in αMEM. Tissues in enzyme solution were placed in a Roto-Therm^™^ Plus Incubated Rotator (Benchmark Scientific, H2024) at 37°C while rotating in combination with oscillations for an hour. αMEM supplemented with 10% Fetal Bovine Serum (FBS) and 1% Pen-Strep was added to the enzyme solution at a 2:1 ratio to inactivate the proteases in the digestive solution. The complete tissue solution was strained through a 70-micron filter, centrifuged, and resuspended in αMEM supplemented with 10% FBS, 1% Pen-Strep, and 55 μM 2-Mercaptoethanol (Thermo Fisher Scientific, 21985023). The isolated tenocytes were seeded in a T-75 flask (Corning) coated with 1 μg/cm^2^ Fibronectin (Sigma-Aldrich, F1056).

Cell culture was performed under standard conditions (37°C, 5% CO_2_, 95% humidity) with a media change after 4 days post-plating and then every other day until sub-confluence was achieved. Cells were passaged at a 1:4 split ratio using 0.25% trypsin/0.02% EDTA solution (Sigma-Aldrich, 25200056). After passage 3, the cells were cryopreserved in Recovery^™^ Cell Culture Freezing Medium (Thermo Fisher Scientific, 12648010) for upcoming experiments. At that point, cells were thawed and cultured in a T-175 flask under standard conditions in αMEM supplemented with 20% FBS for 24 hrs. Media was changed to αMEM supplemented with 10% FBS for 3 additional days, after which the media was changed to αMEM supplemented with 5% FBS. Tenocytes were trypsinized at day 5 after reaching ~ 80% confluency for suspension in the collagen hydrogels.

#### Monocytes/Macrophages

All blood samples were collected in EDTA pre-coated tubes from healthy volunteers under an IRB-approved protocol (STUDY0004777) and processed immediately after collection. PBMCs were isolated from whole blood by density separation over a solution of 1-Step^™^ Polymorph (Accurate Chemical & Scientific Co., AN221725) at 500×g for 30 min without brakes. The white buffy PBMC layer was then washed twice in Wash buffer (HBSS without Ca^2+^ and Mg^2+^ with 10mM HEPES and 5mg/ml BSA) and centrifuged at 350×g for 7 min without brakes to remove platelets. Red blood cells were lysed by exposing the washed PBMC layer to 1/6x PBS for 1 min, followed by 4x PBS for 1 min, at a 3:1 ratio, and spinning at 350×g for 7 min. PBMCs were resuspended in Wash buffer and spun down at 350×g for 7 min to remove any remaining PBS. PBMCs were resuspended in an Isolation buffer (1XDPBS without Ca^2+^ and Mg^2+^ with 2mM EDTA and 1 mg/ml BSA). CD14^+^ Monocytes were isolated from PBMCs by positive magnetic enrichment using the QuadroMACS Starting Kit (LS) (Miltenyi Cat# 130–091-051) using the manufacturer’s protocol.

#### Cell-laden hydrogel

For TC-hydrogel monocultures, P3-P6 tenocytes were suspended at 500,000 cells/ml in collagen hydrogel (TeleCol^®^-3, Advanced Biomatrix, 5026). The cell suspension was introduced into the collagen stock solution at a 1:18 ratio to achieve a final hydrogel concentration of 2.6 mg/ml. The hydrogel mixture was pipetted into the bottom channel and supplemented with 200 μl of X-VIVO^™^ 10 media. At D_0_, the media in the reservoir was replaced with ± TGF-β1 supplemented X-VIVO^™^ 10 (10 ng/ml TGF-β1 (R&D Systems, 240-B-002), 20 μg/ml plasminogen (Haematologic Technologies, HCPG-0130), and 50 ng/ml of tPA (Fisher Scientific, NBP25955350).

For TC/tMφ co-cultures, freshly isolated monocytes were suspended in TeleCol^®^-3 with tenocytes at a ratio of 1:7. The hydrogel was pipetted into the bottom channel and 200 μl of X-VIVO^™^ 10 media supplemented with 20 ng/ml M-CSF (PeproTech, 300 – 25) was added to induce monocyte polarization into naïve macrophages. M-CSF supplemented media was entirely replaced on D_− 4_ and D_− 2_ and ½ replaced on D_− 5_ and D_− 3_.

#### Endothelial Cells

Pooled Human Umbilical Vein Endothelial Cells (HUVECs) were purchased from LONZA (C2519A) and maintained in a T-75 flask at 37°C, 5% CO_2_, 95% humidity in EGM-2^™^ media (LONZA, CC-3162). HUVECs were used between passage number 3–7 as recommended by the supplier and cultured according to the supplier’s protocol.

#### Quad-Culture hToC Assembly

The hToC top component was assembled as described above. To prevent bubble formation at the interface of the trench of the porous membrane and the tissue hydrogel in the bottom channel, the membrane trench was backfilled with collagen solution and was then placed in the incubator at 37°C, 5% CO_2_, 95% humidity for 20 min to allow the collagen hydrogel to crosslink. After 20 min, the top components were removed from the incubator, and the reservoirs were attached and filled with 225 μl of EGM^™^-2 media. The back-filled top components were placed onto the media-filled reservoirs to prevent the collagen hydrogel in the trench from drying out. The well-side of the porous membrane was then coated with 0.17 mg/ml fibronectin (Sigma, F1141) for 1 hr at room temperature to facilitate cell adhesion. Expanded HUVECs were plated into the 100 μl top component well at a 40,000 cell/cm2 density. Cells were allowed to settle for 3 hours before rinsing with EGM^™^-2 media to remove non-adherent cells. HUVECs were incubated for 24 hours in the top component alone before combining with the bottom component containing the TC//tMφ hydrogel. EGM^™^-2 media was replaced with X-VIVO^™^ 10 media in the top component well after assembly to establish serum-free conditions during the experimental timeline.

For tri- and quad-culture assembly, the top component is adhered to the bottom channel to form a tendon-endothelial barrier. Prior to assembly, the bottom channel cultures must be ready for assembly (24-hr culture for TC only and 6-day culture for TC/tMφ co-cultures), the ECs in the top component must have formed a confluent monolayer (expected after 24-hrs), and CD14^+^ monocytes freshly isolated. After full assembly of the hToC, the bottom channel was filled with 100 μl of X-VIVO^™^ 10 media ± TGF-β1. Freshly isolated CD14^+^ monocytes suspended at 100,000 cells/ml in 100 μl of X-VIVO^™^ 10 media were added to the top component well containing the EC monolayer. For all rapamycin experiments in this study, a 10 ng/ml rapamycin solution was added to the EC well at D0. The fully assembled hToC was then placed in an incubator and cultured at standard conditions. Culture supernatants from the top well and bottom channel were sampled from 24- and 72-hour devices for cytokine analysis. At the end of the experiments, EC monolayers and hydrogels were fixed or processed for immunofluorescence or alternative downstream assays at 24 and 72 hours, respectively.

#### Live-Stain Imaging and Quantification of Macrophage Transmigration in hToC

TC, tMφ, and cMφ were live-stained using the ViaFluor SE Cell Proliferation Kit (Biotium, 30068-T) before seeding into the hToC and imaged at 3-, 24- or 72- hrs, respectively. For each image, a z-stack was taken every 10 minutes over 45 minutes, starting ~ 50 μm on top of the porous membrane and 600 μm under the porous membrane to capture cMφ migration through the EC monolayer and into the bottom channel compartment. The hToC was placed on an incubation stage at standard conditions for the entire imaging session to prevent cell death during imaging.

#### Luminex Multiplex Immunoassay

Human Luminex Discovery Assay (R&D Systems) was used for the quantification of selected human cytokines and chemokines: MCP-1, CCL3, CXCL10, IL-1β, IL-6, IL-10, IL-17, and TNF-α in cell supernatants collected from the top well and bottom channel (~ 110μl) and frozen immediately at −80°C. The measurements were performed according to the manufacturer’s specifications using a Luminex 200 Instrument. The experiments were performed with at least three biological replicates per condition, each performed in duplicate.

#### Immunostaining and Fluorescence Microscopy

Tendon constructs were rinsed with 1× PBS, fixed in 4% Paraformaldehyde (PFA) for 10 min, and then washed 2 × 5 min in PBS. Cells were then permeabilized with 0.5% Triton X-100 (Amresco, 0694) for 10 min and rinsed. The constructs were blocked in 1% Bovine Serum Albumin (BSA) (Cell Signaling Technology, 9998) solution for 30 min at Room Temperature (RT) and incubated with the primary antibody solutions (Supplemental Table) diluted in 0.1% BSA or PBS overnight at 4°C. The respective secondary antibody (Supplemental Table 2) used at the manufacturer’s recommended dilution was diluted in 0.1% BSA or PBS and incubated for 1 hr at RT. Hoechst stain was then diluted in PBS and incubated for 10 min at RT. Alternatively, hydrogels were fixed in 4% PFA, dehydrated, removed from the channel and embedded in paraffin. The blocks were sectioned at 10 μm thickness. For IHC, deparaffinized and rehydrated sections were incubated in 10 mM sodium Citrate (pH 6.0) for 1 hr at 65°C for antigen retrieval. Slides were rinsed with ddH_2_O for 5 min followed by 3 × 4 min washes in PBS and 2 × 4 min washes in PBST (PBS + 0.1% Tween-20). Slides were blocked for 1 hr in 5% BSA diluted in PBST and incubated with primary antibodies (Supplemental Table) at 4°C overnight. After primary antibody incubation, the slides were washed 3 × 10 min in PBS and then incubated with respective secondary antibodies diluted in 5% BSA diluted in PBST. Slides were then rinsed 3 × 5 min in PBST, followed by a 2 × 5 min wash in PBS. Sections were cured with ProLong^™^ Diamond Antifade Mountant with DAPI (Thermo Fisher, P36962) overnight at RT in the dark. Clear nail polish was used as a sealant the following day, and slides were stored at 4°C until imaged.

For ICAM-1 and VCAM-1 expression in EC monolayers, live stains were used (Supplemental Table 1). This required adding the primary antibodies diluted in X-VIVO^™^ 10 media and incubating at standard conditions for 15 min before fixing. Cells were then blocked with blocking buffer (5% BSA + 0.1% Triton X-100) and incubated at RT for 10 min. For ICAM-1 and VCAM-1, secondary antibodies and Hoechst stain were added in blocking buffer and incubated for 1 hr at RT. In the case of VE-Cadherin and PECAM-1 staining, primary antibodies were diluted in blocking buffer and incubated for 1 hr at RT after the blocking step. After rinsing the well, the secondary antibody + Hoechst stain diluted in blocking buffer were added and incubated for 1 hr at RT. All samples were stored in a humidified chamber at 4°C and protected from light until imaging. All images were acquired using a Dragonfly Spinning Disk Confocal System (Andor, Belfast, UK) at the University of Rochester High Content Imaging Core.

#### RNA Extraction from Collagen Construct and Bulk RNA-Seq

The cell-laden hydrogels (n = 6 samples per group, with each sample consisting of 2 pooled hydrogels) were harvested from the hToC bottom compartment immediately following the experiment. The hydrogel was digested to release the cells by adding 500 μl of 1 mg/ml (215 units/mg) of collagenase I (Thermo Fisher, 17018029) diluted in X-VIVO^™^ 10 medium and stored at standard conditions for 45 min. Total RNA was isolated from released cells using the RNeasy Plus Micro Kit (Qiagen, 74134) per manufacturer recommendations. To isolate RNA from the EC monolayer, 100 μl of Lysis Buffer was added to the top component well for 10 min. The EC lysate was immediately processed following the manufacturer’s protocol. The RNA concentration was determined via NanoDrop 1000 spectrophotometer (NanoDrop, Wilmington, DE), and RNA quality was assessed with the Agilent Bioanalyzer (Agilent, Santa Clara, CA). Messenger RNA isolation and next-generation RNA sequencing analysis were performed by the University of Rochester Genomics Core.

#### Bioinformatics and statistical analysis of DEGs

All bioinformatic analysis was performed with original RStudio scripts (*R Core Team (2022). R: A language and environment for statistical computing. R Foundation for Statistical Computing, Vienna, Austria.URL* https://www.R-project.org/). Using DESeq2, gene expressions were compared between the human (uninjured and tenolysis from Zheng et al^16^) and hToC samples (vascular and tissue separately). To correct for differences attributed to batch effects, all count data was batch adjusted using *ComBat-seq*. The Wald test was used to generate P values and log_2_(fold changes). Genes with P < 0.05 and absolute log_2_(fold changes) > 1 were selected as differentially expressed genes for each comparison. Heatmaps, dot plots, and enrichment maps were performed using the Complex Heatmap Visualization and Enrichplot packages. Pathway analysis for Gene Ontology (GO), Kyoto encyclopedia of genes and genomes (KEGG) and Reactome were performed using their respective homo sapien database in RStudio. The differentially expressed genes obtained from RNA sequencing were ranked from highest to lowest expression and uploaded to GSEA software.

## Data Availability

The RNA-seq raw and processed data were deposited in the Gene Expression Omnibus under accession GSE244543 and GSE246391. All data generated during this study are available from the corresponding author upon reasonable request.
